# Automatic instance segmentation of orchard canopy in unmanned aerial vehicle imagery using deep learning

**DOI:** 10.3389/fpls.2022.1041791

**Published:** 2022-12-01

**Authors:** Weirong Zhang, Xuegeng Chen, Jiangtao Qi, Sisi Yang

**Affiliations:** ^1^ Key Laboratory of Bionic Engineering, Ministry of Education, Jilin University, Changchun, China; ^2^ College of Biological and Agricultural Engineering, Jilin University, Changchun, China; ^3^ College of Mechanical and Electrical Engineering, Shihezi University, Shihezi, China; ^4^ Institue of Scientific and Technical Information of Jilin, Department of Science and Technology of Jilin, Changchun, China

**Keywords:** deep learning, instance segmentation, orchard, canopy, convolutional neural network, unmanned aerial vehicles

## Abstract

The widespread use of unmanned aerial vehicles (UAV) is significant for the effective management of orchards in the context of precision agriculture. To reduce the traditional mode of continuous spraying, variable target spraying machines require detailed information about tree canopy. Although deep learning methods have been widely used in the fields of identifying individual trees, there are still phenomena of branches extending and shadows preventing segmenting edges of tree canopy precisely. Hence, a methodology (MPAPR R-CNN) for the high-precision segment method of apple trees in high-density cultivation orchards by low-altitude visible light images captured is proposed. Mask R-CNN with a path augmentation feature pyramid network (PAFPN) and PointRend algorithm was used as the base segmentation algorithm to output the precise boundaries of the apple tree canopy, which addresses the over- and under-sampling issues encountered in the pixel labeling tasks. The proposed method was tested on another miniature map of the orchard. The average precision (AP) was selected to evaluate the metric of the proposed model. The results showed that with the help of training with the PAFPN and PointRend backbone head that AP_seg and AP_box score improved by 8.96% and 8.37%, respectively. It can be concluded that our algorithm could better capture features of the canopy edges, it could improve the accuracy of the edges of canopy segmentation results.

## 1 Introduction

Technology and equipment for plant protection are crucial for agricultural output (Ouyang et al., 2020). In apple farming, spraying is one of the most important commonly applied canopy management practices, it should be conducted during the stage of apple growth aims to raise the quality of apples and obtain higher yield. However, the low utilization rate of pesticides has been an important factor in the development of China’s application technology (Ru et al., 2015), the utilization rate of pesticides in conventional application methods is only 30%, which not only affects the effectiveness of pest control, but also causes environmental pollution.

The integration of agricultural machinery and information technology is a necessary tool for the development of modern agriculture, which can improve the efficiency of agricultural resources utilization and accelerate the process of agricultural modernization (Chen et al., 2020). With the continuous development of the precision agriculture, remote sensing applications have diversified to include satellite, manned airplanes or unmanned aerial vehicles (UAVs) (Mulla, 2013). UAV images are more easily obtained and it implies lower operational costs, less weather constraints (Rasmussen et al., 2016). UAVs are used for the most autonomous and accurate way to obtain tree’s information.

A considerable amount of research on orchard canopy information focus on the identification and counting of individual trees (Morales et al., 2018; Cheng et al., 2020; Qi et al., 2021). In fact, due to geometric features of plant canopies can offer relevant indicators, individual canopy-related features interested farmers but the most accurate estimations for canopies all mostly based on destructive and costly labour-intensive manual measurements (Gower et al., 1999; Jonckheere et al., 2004; Ma et al., 2017). To overcome these disadvantages, UAV-based imagery in conjunction with computer vision methodologies have become widely used on the research of tree extraction (Nyamgeroh et al., 2018; Durfee et al., 2019).

Brede et al. (2017) concluded that UAV-borne laser scanning(ULS) has the potential to perform comparable to Terrestrial Laser Scanning for estimating forest canopy height. ULS combines the strengths of above and under canopy surveys, the results showed that in easy forest stand conditions, the performance of ULS point cloud is comparable with the terrestrial solutions (Liang et al., 2019). The UAV-based LiDAR data can be effectively used in canopy cover estimation, individual tree segmentation-based method had the highest accuracy in estimation of canopy cover (R2 = 0.92, rRMSE = 3.5%) can provide references for sustainable management (Wu et al., 2019). Laser scanning data of stem curve was obtained by using UAV. Novel data processing algorithms were applied for the point clouds to extract the stem curves and diameters at breast height (Hyyppa et al., 2020). However, these methods using LiDAR represents an important limitation for costly.

There exist other methods that use multispectral cameras to descriptor such as canopy shape, crown contour and canopy volume. In order to estimate tree height, Wu et al. (2020) compared several methods. Height estimations of mango and avocado trees were compared to canopy metrics obtained from Airborne Laser Scanning (ALS) and UAV-based RGB and multi-spectral photography. Chang et al. (2020) used UAV-based multispectral pictures to compare the canopy shape and vegetation indicators of range trees. The findings revealed a strong correlation between tree height and canopy volume measured from the ground and by UAV. Gallardo-Salazar et al. (2020) analyzed included different vegetation indices estimated with a high-resolution orthomosaic and obtained total height and the crown diameter of individual trees, the consistency of the the normalized-difference vegetation index(NDVI) as the most recommended to evaluate productivity results for its application in the field.

When focusing on RGB images, a large number of studies of tree phenotype in orchards can be found. Using image processing techniques, Yıldız et al. (2020) determined the canopy area of apple trees. Regression analysis employed both circular and elliptical calculating techniques. Using a local-maxima-based technique on UAV-derived Canopy Height Models (CHMs), Mohan et al. (2017) assessed the applicability of low-altitude visible light image and structurefrom-motion (SFM) algorithm). To distinguish between overlapping tree crown projections, Ponce et al. (2021) developed a novel method for crop tree identification using image analysis techniques, doing away with the usage of vegetation indices and machine learning-based approaches. The aforementioned methods, however, are likely to have a low fidelity for interlaced orchards. Cheng et al. (2020) provided a segmentation approach for mingled fruit tree canopies with irregular forms that makes use of a Gaussian Mixture Model and XGBoost to accurately recover the individual apple and cherry trees from mingled canopies.

In recent years, the performance of the CNN network in detecting complicated phenomena has been excellent due to the accessibility of massive datasets and the ongoing advancement of GPU processing power. A growing variety of artificial intelligence algorithms have been used in horticulture research and remote sensing for agriculture (Kamilaris and Prenafeta-Boldú, 2018; Zhou et al., 2020; Yang and Xu, 2021; Qi et al., 2022). Mo et al. (2021) proposed a deep learning-based instance segmentation method YOLACT of litchi trees. The boundary and location information of the canopy have been obtained by using the Digital Orthophoto Map (DOM). A Convolutional Neural Network (CNN) based on the Deeplab v3+ architecture was used to detect full-grown isolated Mauritia flexuosa palms, and has achieved better performance than those of other CNN networks used for performance comparison (Morales et al., 2018). Lou et al., (2022) used thrss widely object detection methods such as the Faster region-based CNN (Faster R-CNN) (Ren et al., 2015), You Only Look Once version 3 (YOLOv3) (Redmon et al., 2018), and single shot detection (SSD) (Liu et al., 2016) to identify tree crowns and their widths in two loblolly pine plantations, respectively.

Due to unsystematic tree branches overlapping and shadows, the accuracy of the deep learning-based image segmentation algorithms needs to be improved. In horticultural computer vision, however, it has always been challenging to detect the boundary of tree canopies.

In this regard, we offer an innovative technique for precisely segmenting the borders of apple trees using aerial photos taken with RGB cameras placed on UAVs. This approach aims to address the issue of incorrect segmentation of tree canopies in dense orchards with complex backgrounds, including branches and shadows. Firstly, RGB images were processed in DJI Terra software to yield a Digital Orthophoto Map (DOM), then DOM was sliced into smaller images for training the deep learning model. Second, the feature of canopy instances was extracted using the PAFPN (Liu et al., 2018) as backbone neck and PointRend (Kirillov et al., 2020) as a new backbone head based on the instance segmentation of the Mask R-CNN (He et al., 2017) framework. Our method is called MPAPR R-CNN. This segmentation is eventually combined into a miniature orchard map, with each little picture containing the canopy’s pixel count by segmentation network. The whole system was put to the test in an apple orchard, and the comparison experiment findings showed how well it works for identifying apple tree canopy.

## 2 Materials and methods

### 2.1 Study area

**Figure 1:**
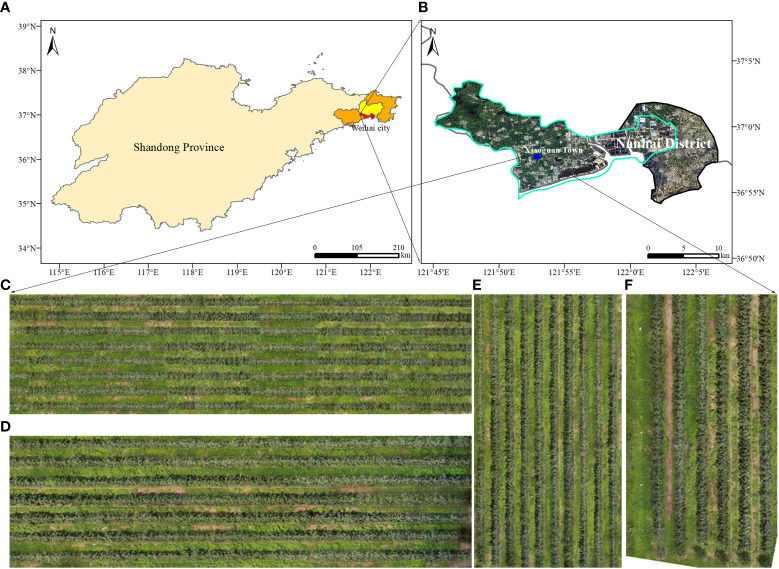
Test location of image capture. **(A, B)** The location of the experimental orchard. **(C-E)** Digital orthophoto maps for training and **(F)** for testing in canopy detection.

As shown in **Figures 1A, B**, the study was conducted during the summers of 2022 at the JingXiang Orchard in Weihai City, Shandong Province, China. The location is characterized by a temperate monsoon climate, with average annual precipitation of 400–600 mm and an average effective temperature during the study period (July–August) of 28°C. The local climate is perfect for the cultivation of apples.

The orchards under study are high-density planting patterns with a 3.5-meter route between rows and a tree spacing of 0.8 meters. It should be emphasized that the planting and management model adheres to the region’s suggested production techniques. 'Four location DOMs containing apple orchards of different ages were used for canopy identification in this paper, where C, D and E of **Figure 1** were used as training for the model, while Map F was used as a test map for the model training results.

### 2.2 Unmanned aerial vehicle image collection

**Figure 2:**
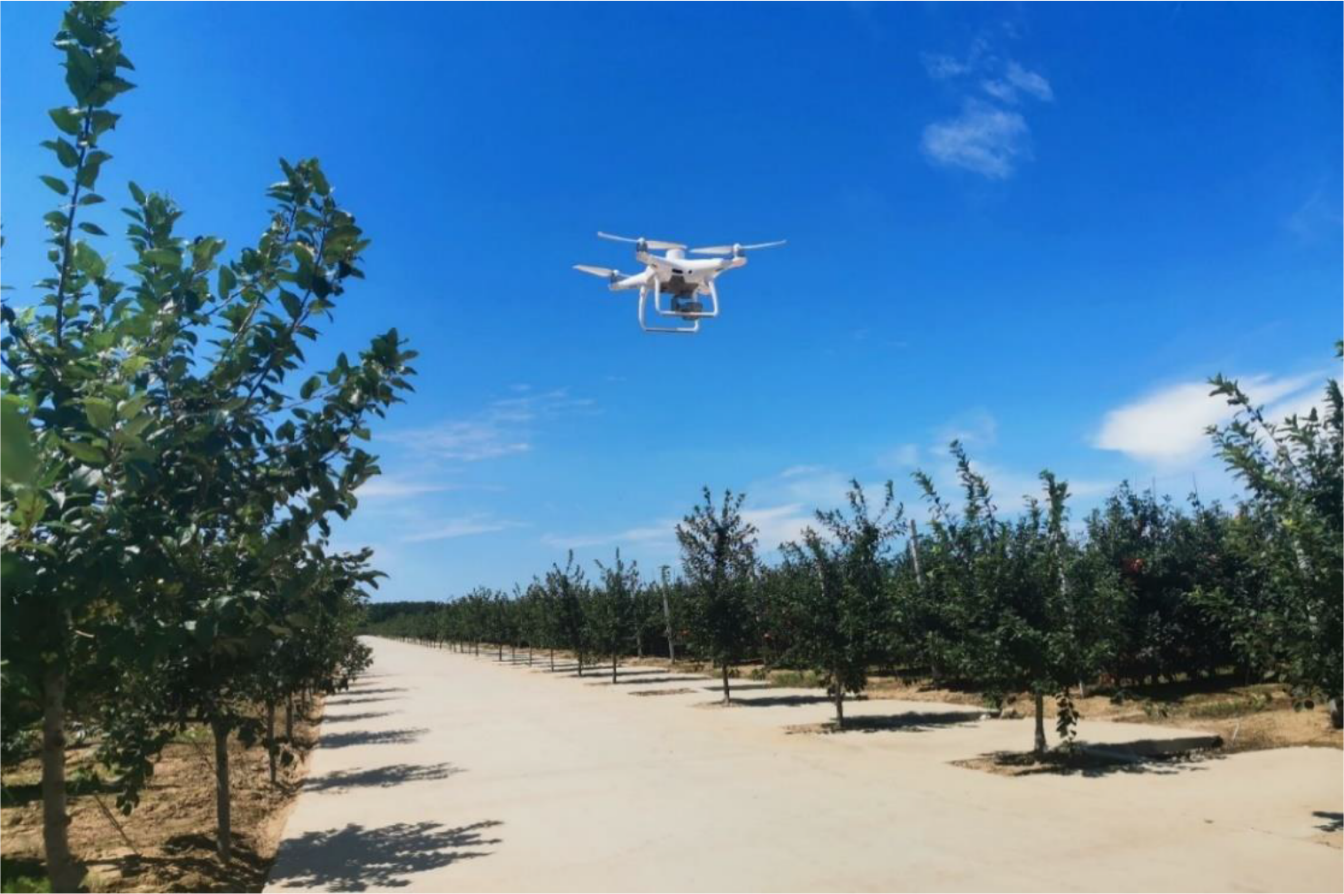
The DJI P4 Multispectral.

Apple trees were captured with the DJI Phantom 4 Multispectral (P4, SZ DJI Technology Co., Ltd., Shenzhen, Guangdong, China). The P4 is employed because it can be programmed to fly independently, and the collected visible images can be processed to generate orthophoto images, or other drones equipped with low-cost RGB visible light can be used. For multispectral imaging, this UAV was outfitted with one RGB sensor and five monochrome sensors, which have six 1/2.9-inch CMOS, including one color sensor for visible imaging and five monochrome sensors for multispectral imaging. Individual sensors have 2.08 million effective pixels (2.12 million total pixels). **Figure 2** depicts the takeoff of a drone for data collection.

The purpose of this paper is to solve the problem of UAV canopy image segmentation in complex backgrounds, and we selected the area where weeds are most abundant for UAV flight. To minimize any shadow effects, the flight was conducted during sunny or cloudy weather conditions at high noon, with very light winds, between approximately 11:30 am and 12:30 pm. The DJI GO Pro software was used to set up the flight for autonomous management. The pictures have an 80% mean forward overlap and a 70% mean side overlap. The aircraft was maintained at a cruise speed of 2 m/s an altitude of 15 m above ground and during the flight. The aircraft maintained a cruise speed of 2 m/s during flight at 15m and 20m altitude, while the ground sample distance (GSD) was 0.79cm/pixel and 1.06cm/pixel, respectively.

### 2.3 Canopy segmentation framework

**Figure 3:**
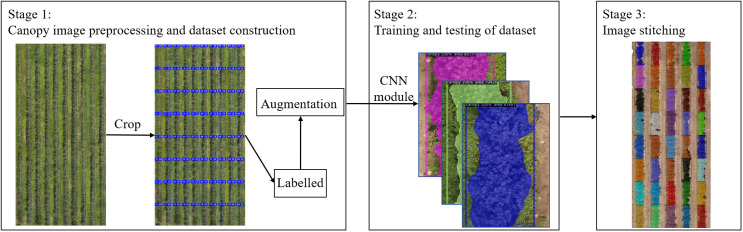
Canopy segmentation framework.

We first summarize the whole process of the proposed framework for detecting orchard canopy and then discuss in detail each phase of the model. As shown in **Figure 3**, the framework consists of three major parts: (1) image dataset construction and preprocessing; (2) training and inference and (3) image stitching.

#### 2.3.1 Image preprocessing and dataset construction

In this part, DJI Terra software was used to convert the UAV canopy images into DOM. Since the resolution of DOM is too large, the images need to be cropped to meet the appropriate size required for computer operation, then we use Labelme software for annotation, and then perform image enhancement to generate the image dataset of orchard canopy for defect and segmentation model training and testing.

#### 2.3.2 Training and testing of datasets

**Figure 4:**
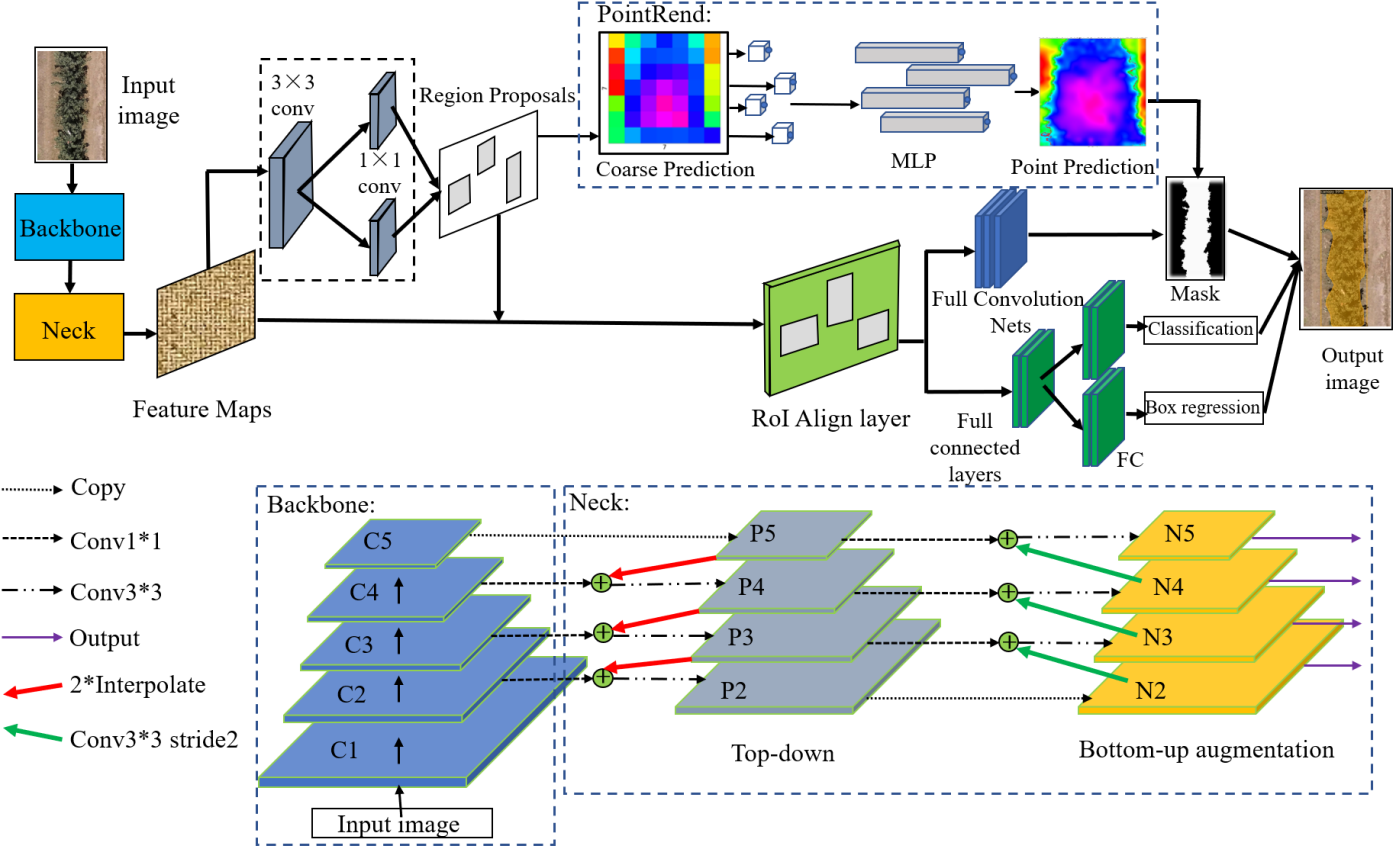
Canopy segmentation model base on Mask R-CNN. * indicates that the height and width of the convolution kernel matrix are multiplied.

In this section, we proposed to design our framework based on Mask R-CNN. In order to fit the tree canopy detection and segmentation task, as in **Figure 4**, we introduced the PAFPN and PointRend into the original architecture. The proposed model can obtain enhanced features with both rich context information and edge information, leading to better performance of canopy detection and segmentation results. In addition, considering the shape characteristics of canopy in cropped image, we modifed the aspect ratios of anchor boxes in the RPN network. Specific network design will be described in the later section.

#### 2.3.3 Image stitching

After the deep learning model had been trained and the test photos had been post-identified, this segmentation is eventually combined into a miniature orchard map using Adobe Photoshop CC 2019 software. With each little picture containing the canopy’s pixel count by segmentation network, the orthophotography can be used to provide application recommendations to variable application machinery.

### 2.4 Canopy segmentation method

#### 2.4.1 Image preprocessing and dataset construction

Using DJI Terra software, over 500 photos taken by the P4 UAV of the experimental regions every flight were photogrammetrically processed to create the RGB DOM. Through the training of a large amount of data, the model based on deep learning can achieve great prediction results for complex classification and detection tasks.

DOM resolution is too huge for processing, especially for deep-learning-based methods, thus the high-resolution picture was chopped using the Adobe Photoshop CC 2019 software slicing tool, and the DOM was ultimately divided into 500 little pixel images of 450*600. To boost the variety of the canopy photos for the deep learning network, techniques including noise addition, random blurring, panning, vertical mirroring, and diagonal flipping were applied. A final dataset of 2000 enhanced canopy images were produced as a consequence of the data augmentation strategies, which also expedited the dataset’s creation, improved the resilience and generalization of the model training, and decreased the likelihood of overfitting. Finally, we divided the training and validation sets for 2000 images in the ratio of 8:2.

#### 2.4.2 Architecture of mask R-CNN

Mask R-CNN is a classical image segmentation algorithm that detects target objects in an image and marks the outline of the object region, extracting the relevant pixels for area calculation. Faster R-CNN for target recognition and a Fully Convolutional Network (FCN) for semantic segmentation are combined to create Mask R-CNN. The Faster Convolutional Network (FCN) is utilized for mask prediction, boundary regression, and classification based on the target discovered by the Faster R-CNN. These include a feature extraction layer using ResNet/ResNeXt as the convolutional backbone, a region suggestion network (RPN), bilinear interpolation (ROIAlign), and fully connected FC and FCN.

The selected region of interest (RoI), after mapping to the feature map, is further pixel-corrected by the ROIAlign layer. The resultant feature map is delivered to a region proposal network (RPN) to create positive and negative samples. Because the picture enhancement in this investigation did not involve a 90-degree rotation to increase the dataset, the orientation of the canopy photographs in this study all stretched along the vertical direction. The initial model was enhanced by balancing the distribution of various picture forms and constructing anchor points with three distinct scales of 0.3, 0.5, and 1 in aspect ratio to increase the identification and segmentation accuracy of the canopy.

#### 2.4.3 Feature extraction network

**Figure 5:**
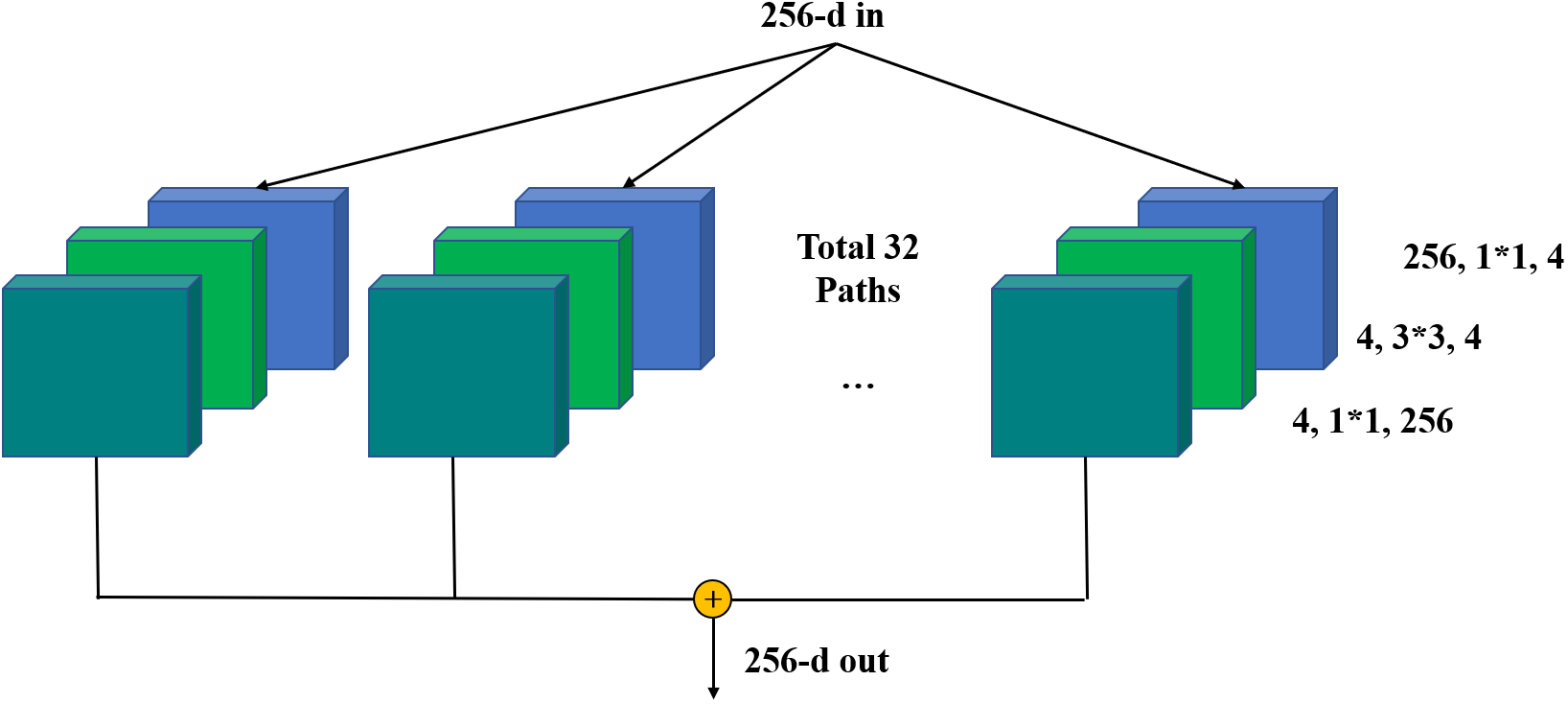
Backbone Network-ResNeXt. * indicates that the height and width of the convolution kernel matrix are multiplied.

To achieve more effective detection, ResNeXt is regarded as the backbone network for feature extraction of the input image. ResNeXt is built on ResNet modular structure and incorporates the high recognition performance of split‐transform‐merge in Inception. The right side of **Figure 5** shows the structure of each basic unit.

In **Figure 5**, ResNeXt uses multiple convolution modules to perform feature extraction from bottom-up, and group convolution uses the same topology on different input channel paths. By using cardinality as a super parameter, it’s able to achieve a more efficient network. For a 256-dimensional input with cardinality of 32, the network encodes 256 channels into 4 channels, and the features are extracted in 32 different embedding spaces by 32 different groups consisting of continuous 1 × 1 conv, 3 × 3 conv, and 1 × 1 conv.

#### 2.4.4 Feature fusion network

In multilayer convolutional neural networks, features at shallow layers are usually more representative of edge morphology, which is crucial for accurate pixel classification and instance segmentation (Kong et al., 2016), and it is precisely the determination of instance edges that is most important for segmentation of crown images. Specially, we adopt a path augmentation feature pyramid network (PAFPN) to enhance the feature hierarchy with rich low-level features by adding a bottom-up path augmentation module and a feature fusion operation module.

**Figure 6:**
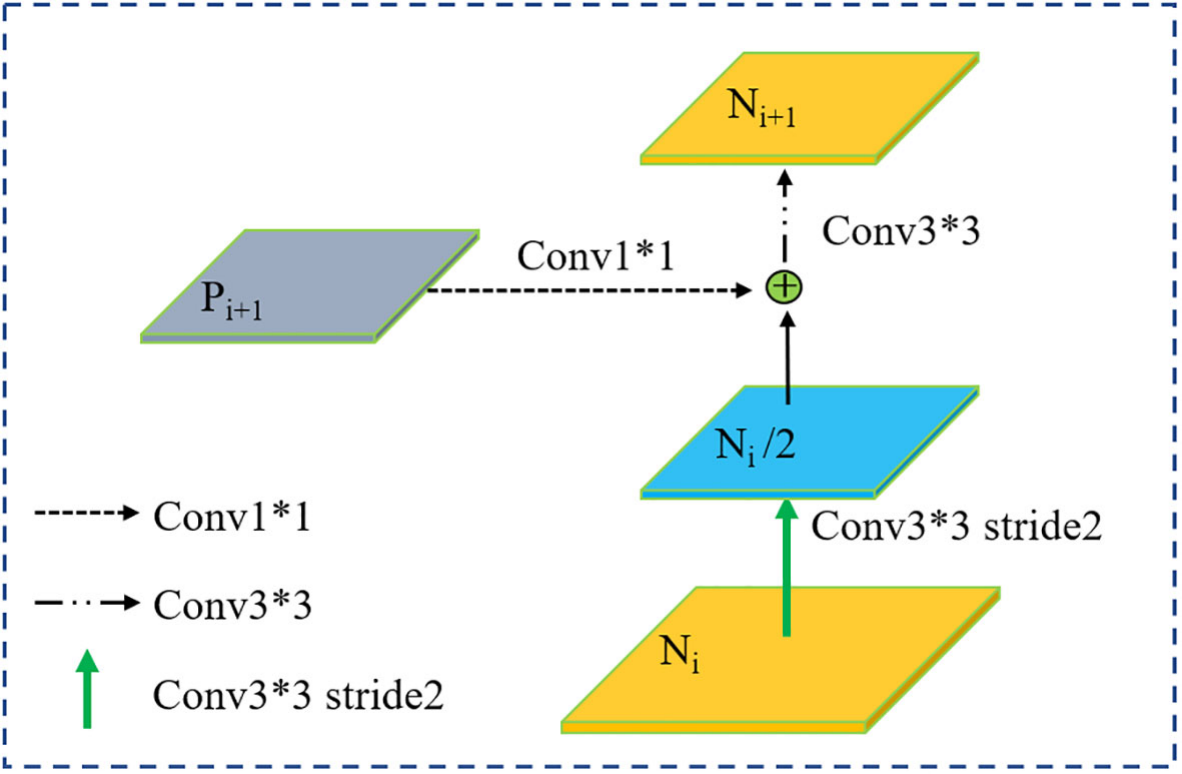
Bottom-up path augmentation. * indicates that the height and width of the convolution kernel matrix are multiplied.

The part of Neck in **Figure 6** shows the PAFPN module in details. Each cube represents a corresponding feature tensor. In the original ResNeXt-FPN backbone network, features are extracted from the final convolutional layer of conv1–conv5 parts of ResNeXt101, which are called C_1_, C_2_, C_3_, C_4_ and C_5_ in this paper. Based on the bottom-up network architecture, the feature extraction layers compute hierarchical feature maps. Feature maps generated by FPN are represented by P_2_, P_3_, P_4_, P_5_.

The feature maps of the added bottom-up path augmentation module are represented as N_2_, N_3_, N_4_ and N_5_ corresponding to P_2_ to P_5_. The concrete operations for bottom-up path augmentation module are illustrated in **Figure 6**. Firstly, N_i_/2 is obtained by a 3 × 3 convolutional layers with stride 2 to down-sampled, where the size of N_i_/2 is reduced by a factor of two. Then the down-sampled feature map is concatenated with P_i+1_. At last, the fused feature map goes through another 3 × 3 convolutional layer to generate new feature map N_i+1_. Then, feature fusion operations are carried out to incorporate higher level feature maps to the lower-level ones for contextual feature fusion.

#### 2.4.5 Optimized boundary feature based on PointRend technique

As objects have irregular boundaries, most segment methods can classify pixels inside the object accurately but pay less attention to the accuracy drop caused by upsampling on the edge of the object increases the loss of prediction. Image segmentation tasks of original Mask R-CNN focus on regular grids to classify each pixel in the image has an obvious drawback of shivering or over-smoothed edges of segmentation, which makes the boundary of the mask unsatisfactorily and greatly undermines the accuracy of canopies edge segment.

As a result, to address this issue, we employed a high-quality PointRend module to recover clear and sharp mask edges. This module can adaptively choose a non-uniform set of points by a subdivision strategy to densely sample and label the boundary pixels while minimizing the indistinct segmentation results. Point selection, point-wise feature extraction, and point head make up the three primary components of PointRend.

The point selection module chooses suitable sampling points flexibly and adaptively to predict to avoid excessively computed pixels, and focuses on the points located near object boundaries.

**Figure 7:**
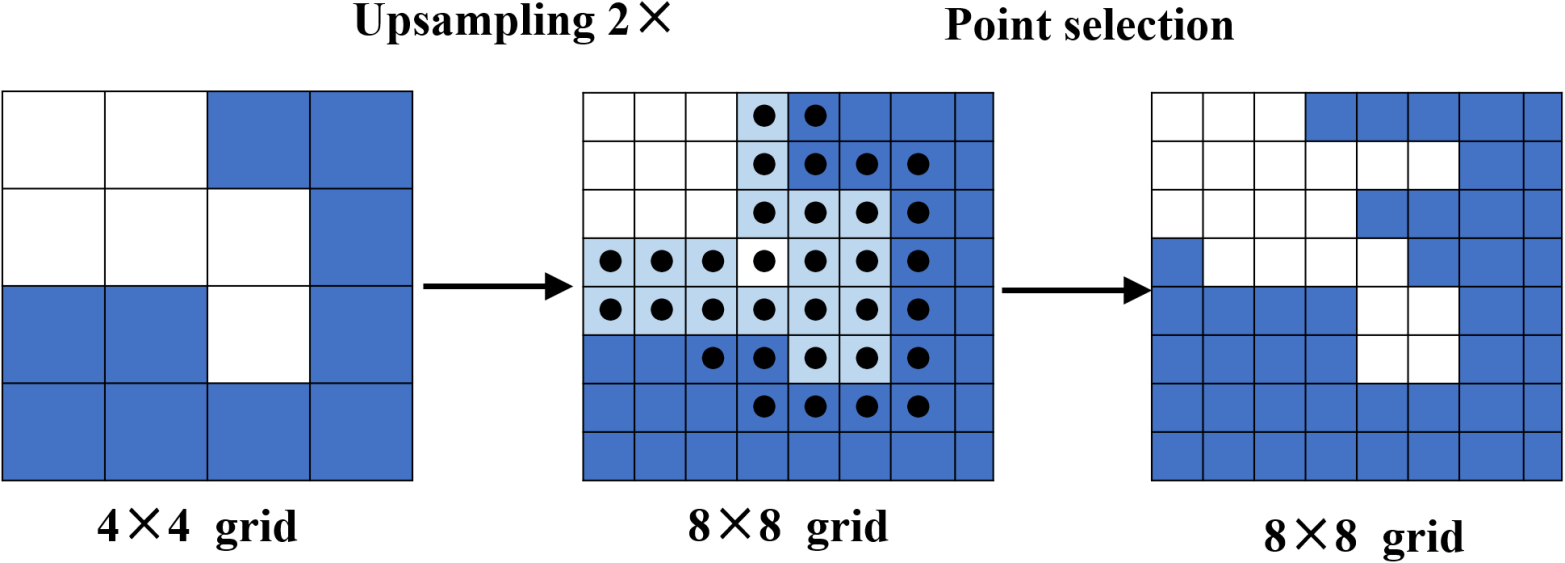
The strategy of the point selected.

After the target segmentation model output feature map as the initialization output map of the PointRend model, the strategy of point selection is to render the output image in a coarse-to-fine manner. The first prediction is the coarsest and is performed on the point of a regular grid. As shown in **Figure 7**, in each iteration, the points on a regular grid from the low spatial resolution feature map will be predicted coarsest first. The output result is sampled up by bilinear interpolation to achieve the denser feature prediction map. Then on the high-resolution segmentation map, where the N most uncertain points are concentrated in the edges, the confidence interval is [0,1] close to 0.5. Points are selected by Equation (1). Once N points are selected, point-wise feature extraction is performed. These N points are the points that are finally filtered out for re-confirmation. And so on, iterating step by step to obtain the final segmentation map with the target resolution.


(1) $$n_{i}^{*}=\text{arg}\underset{{\text{n}}_{\text{i}}}{\text{min}}\left|\text{p}\left({\text{n}}_{\text{i}}\right)-0.5\right|$$

where p(n_i_) is the probability for point n_i_ belonging to the binary mask; $n_{i}^{*}$ is the selected point.

For training, the point selection strategy is a random sampling-based selection strategy. First, kN candidate points(k>1) are randomly sampled from the feature map to address the uncertain regions while keeping a uniform distribution. Then kN points are sorted while estimating the uncertainty. The most uncertain βN points are selected, where β ∈ [0,1]. These points are concentrated in the most uncertain area, such as road boundaries. Last, the surplus (1−β) N points are distributed from a uniform distribution.

The coarse prediction and fine-grained features are combined to create the point-wise feature of the selected points extraction module. Regarding fine-grained features, bilinear interpolation is used to extract the finely detailed segmentations from each point chosen from the sort in the feature map to display the fine segmentation details. These segmentations are then stored in feature vectors, which contain fine-grained features.

Fine-grained features may contain only relatively low-level information and do not obtain specific region information, but the coarse predicted feature can provide a more general and globalized context, with a 2-dimensional vector for class-2 prediction at each point in the region.

The pointed head is a simple Multi-layer Perceptron (MLP) used to represent prediction labels based on point-wise features, which can share weights across all points. Moreover, because the MLP predicts a segmentation label for each point, it can be trained by the segmentation loss of a specific task. Note that when the backbone head is replaced with PointRend, the loss of the segment network is increased by loss point, but this does not affect the final segmentation accuracy.

#### 2.4.6 Loss function

The loss function of the Mask R-CNN with PointRend has four components, the classification loss of the bounding box, the position regression loss of the bounding box, and the loss of the mask. The loss function L for each sample ROI in the network is


(2) $$L=L_{\text{box}}+L_{\text{cls}}+L_{\text{mask}}+L_{\text{point}}$$

There are three components: *L*_box_ is the classification calculation loss, *L*_cls_ is the position regression loss of the bounding box and *L*_mask_ is the mask calculation loss. The bounding box loss function, the classification calculation loss, and the mask calculation loss are shown as follows:


(3) $$L_{box}\left(t_{i}^{*},t_{i}\right)={\text{smooth}}_{\text{L}1}\left({\text{t}}_{i}^{*}-{\text{t}}_{\text{i}}\right)$$

(4) $${\text{smooth}}_{\text{L}1}\left(\text{x}\right)=\{\begin{array}{cc} 0.5{\text{x}}^{2} & \left(\left|\text{x}\right|<1\right)\\ \left|\text{x}\right|-0.5 & \left(\left|\text{x}\right|⩾1\right) \end{array}$$

where t_i_=(t_x_,t_y_,t_w_,t_h_) , ${\text{t}}_{i}^{*}=\left({\text{t}}_{x}^{*},{\text{t}}_{y}^{*},{\text{t}}_{\text{y}},{\text{t}}_{w}^{*}\right)$ 。


(5) $${\text{L}}_{\text{cls}}\left({\text{p}}_{i}^{*},{\text{p}}_{\text{i}}\right)=-\log\left[{\text{p}}_{i}^{*}{\text{p}}_{\text{i}}+\left(1-{\text{p}}_{i}^{*}\right)\left(1-{\text{p}}_{\text{i}}\right)\right]$$

where p_i_ represents the probability anchor is predicted to be positive samples, ${\text{p}}_{i}^{*}$ represents the foreground true probability of the anchor point, i.e. a value of 1 when in the foreground and 0 when in the background anchor samples.


(6) $$L_{mask}\left(s_{i}^{*},s_{i}\right)=-\left({\text{s}}_{i}^{*}\log\left({\text{s}}_{\text{i}}\right)+\left(1-{\text{s}}_{i}^{*}\right)\log\left(1-{\text{s}}_{\text{i}}\right)\right)$$

where, *s*_*i*_ represents the probability mask is predicted to be the irightvalue the $s_{i}^{*}$ is the label value of the mask.


(7) $$L_{point}\left(s_{i}^{*},s_{i}\right)=\text{seg}\_\text{loss }+\text{ points}\_\text{loss}$$

where seg_loss represents the cross-entropy loss of the overall pixel point, points_loss represents the cross-entropy loss of the uncertain point.

### 2.5 Algorithm platform

The model training platform is a laptop with Ubuntu 18.04 operating system. The deep learning model in this paper is the Detectron2 framework based on PyTorch, while CUDA 11.1 is used to accelerate the training process. **Table 1** describes the specific environment configuration.

**Table 1:** Image processing unit host hardware and software environment.

Name	Version
CPU	Intel(R) Core(TM)i7-11800H
GPU	NVIDIA Geforce RTX 3050(4GB)
Operating System	Ubuntu 18.04
Computing Architecture	CUDA 11.1
Deep learning Framework	PyTorch1.5.0
Anaconda	Anaconda3(Python3.7.2)

Mask R-CNN employs the alternating optimization training technique. Stochastic Gradient Descent (SGD), a quick and efficient gradient descent technique for convolutional neural networks, is used as the training optimization approach. The maximum number of training iterations is 25000, the number of samples (batch size) used in each trainer is 1, the number of samples in a batch of training samples (one epoch) is 128, and the learning rate decay multiplier (gamma) is 0.2, the learning rate decay is performed after 10000 and 20000 iterations, the number of warm-up iterations is 1000, momentum is 0.9, and weight decay coefficient is 0.001.

### 2.6 Evaluation indicators

To validate the performance of the model, Mean Average Precision (*m*_*AP*_ ) is used as a metric to evaluate the accuracy of the training model. *m*_*AP*_ is an algorithm performance metric used to predict target locations and categories, and refers to the average of the Average Precision (*A*_*P*_ ) of multiple categories, and a higher *m*_*AP*_ value indicates a better model is better. In image segmentation, a curve can be plotted for each category based on the accuracy *P* (Precision) and recall *R* (Recall), and the Average Precision *A*_*P*_ is the area under that curve. Multiple metrics are calculated as follows:


(8) $$P=\frac{T_{P}}{T_{P}+F_{P}}$$

(9) $$R=\frac{T_{P}}{T_{P}+F_{N}}$$

(10) $$A_{P}=\int_{0}^{1}P\left(R\right)\text{d}R$$

where *T*_*P*_ denotes the number of samples correctly predicted as positive, *F*_*P*_ denotes the number of samples in which negative samples are predicted as positive, *F*_*N*_ denotes the number of samples in which positive samples are predicted as negative, and *k* denotes the number of categories; *P* refers to the accuracy rate, which is the proportion of correctly detected samples to all samples actually detected; and *R* refers to the recall rate, which is the proportion of the number of correctly detected samples to the number of samples that should be detected.

## 3 Results

To better validate the performance of the optimized segmentation model, comparative experiments were conducted to demonstrate the detection and segmentation capabilities of the model under different configurations.

### 3.1 Different anchor and backbone

Since the target of detection in this paper is the tree canopy, combined with the canopy growth and the slender characteristics of the collected image data set along the top and bottom directions, the aspect ratio of anchor was adjusted to {1:1, 1:2, 1:3} to suit the canopy detection.

The ResNeXt network is implemented by simply cascading layers of the same structure and implementing a split-transform-merge strategy at each level of the network. Based on the ResNet network structure, a new dimension called “cardinality” is proposed. For canopy detection, we need to verify whether the improvements in the ResNeXt network improve the detection and segmentation accuracy. To test the impact of the improved anchor frame ratio and feature extraction network, we designed a set of comparison experiments. We use the standard metrics average precision (AP, AP50, AP75) to evaluate our results. The results are shown in **Table 2**.

**Table 2:** The detect results of different Anchor and Backbone.

Backbone Network	ImprovedAnchor ratio	ResNeXt	AP_seg			AP_box		
			AP	AP50	AP75	AP	AP50	AP75
Mask R-CNN-FPN			57.24	79.51	79.87	65.14	78.93	77.42
	✔		58.34	79.89	81.32	65.93	80.29	79.18
		✔	58.96	81.26	81.41	66.71	82.56	80.39

✔ indicates that on the basis of the backbone network, add the corresponding module at ✔. The first line is Mask R-CNN-FPN, the second line is Mask R-CNN-FPN+Improved Anchor ratio, and the third line is Mask R-CNN-FPN+ Improved Anchor ratio+ResNeXt.

**Table 2** shows that the improved anchor ratio and ResNeXt both affect the accuracy of the segmentation. Since the canopy distributed along the up-down direction is not rotated by 90 degrees in the data enhancement operation, the detection task of the canopy dataset is better facilitated when the RPN network uses a more elongated anchor frame for the generation of the region suggestion frame. In addition, performing a set of transformations using low-dimensional embeddings by constructing bases in the base block, split-transform-merge strategy can make the deep learning model learn more features. Therefore, improved anchors and ResNeXt were used as part of the Mask R-CNN model for feature extraction and as the base network for subsection 3.2.

### 3.2 Best model configuration

The key differences between our suggested canopy detection and segmentation method and the original Mask R-CNN architecture are two. In order to get feature maps with rich low-level information, we first applied a PAFPN module to the original Mask R-CNN. The second is that we utilized Pointrend to enhance the accuracy of edge segmentation results. Based on Mask R-CNN with better anchor and ResNeXt, we create four distinct network frameworks to extract features in order to test the impact of the new PAFPN and PointRend module, which are represented by RX-FPN (Mask R-CNN+ResNeXt+FPN), RX-PAFPN (Mask R-CNN+ResNeXt+PAFPN), PR-RX-FPN (Mask R-CNN+PointRend+ResNeXt+FPN) and our method(Mask R-CNN+PointRend+ResNeXt+PAFPN), respectively. Our method is called MPAPR R-CNN. Four group experiments are used to detect and segment orchard canopy images in this part.

**Figure 8:**
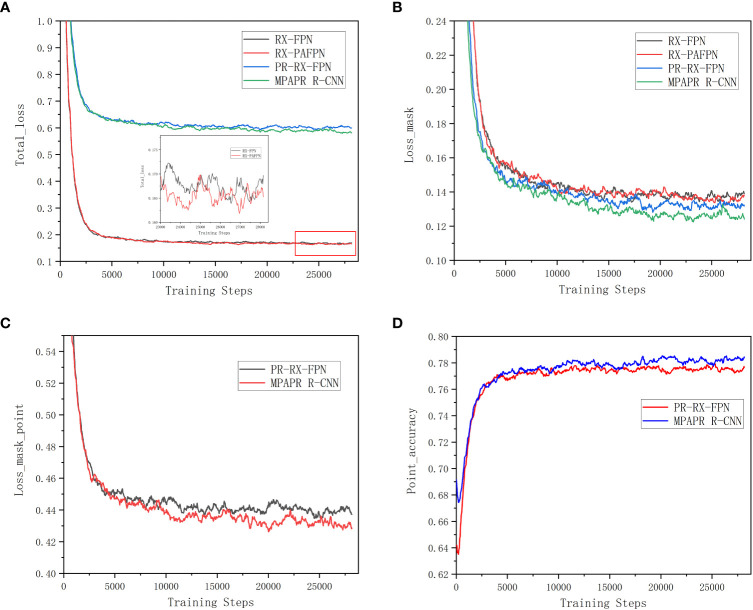
Loss and accuracy curves of several different instance segmentation algorithms in training stages. **(A)** Total loss curves. **(B)** Mask loss curves. **(C)** Mask point loss curves. **(D)** Point accuracy curves.

**Figure 8** compares the loss functions of the four instance segmentation models used in the experimental training phase. In **Figure 8A**, in comparison to RX-FPN and PR-PAFPN, PR-RX-FPN and MPAPR R-CNN have higher total loss due to the training loss function of PointRend contains point loss. However, it is still obvious in **Figure 8A** that the RX-PAFPN with enhanced feature pyramid network has lower loss in RX-FPN and RX-PAFPN without combining PointRend, and similarly, in PR-RX-FPN and MPAPR R-CNN with combining PointRend, the PAFPN module with MPAPR R-CNN model also has lower loss, which indicates that both PAFPNs effectively improve the original FPN network. This can also be seen in **Figures 8C, D**, where PAFPN has a significant effect on the model improvement, firstly, the loss_mask_point is reduced, and secondly, the point accuracy is higher. This further shows the improvement effect of PAFPN on the PointRend model.

For the most important loss_mask of crown segmentation, **Figure 8B** shows that both PR-RX-FPN and MPAPR R-CNN with PointRend module have lower mask loss values than RX-FPN and RX-PAFPN without PointRend module. It indicates that the addition of the PointRend module has a more significant optimization effect on reducing the mask loss of the FPN and PAFPN networks. However, the lowest Loss_mask is the MPAPR R-CNN model with both PAFPN and PointRend.

The AP findings for each of the four networks are displayed in **Table 3**. MPAPR R-CNN outperforms competing methods in terms of AP-seg and AP-box score, which supports its efficacy in identifying canopy images. We can see from **Table 3** that the suggested PAFPN and PointRend algorithm considerably alters the AP score of test outcomes. The AP seg and AP box scores of the RX-PAFPN are increased by 3.18% (from 59.64% to 62.82%) and 1.85% (from 67.61% to 69.46%), respectively, while the value of the AP 50 grows more considerably, improved by 6.28% (from 81.8% to 88.08%) and 6.16% (from 84.4% to 90.56%). The outcomes demonstrate that the PAFPN algorithm may successfully prevent information loss of low-level features and improve the original’s capacity to extract features.

**Table 3:** Comparison of AP results for four different methods.

Network	PAFPN	PointRend	AP_seg			AP_box		
			AP	AP50	AP75	AP	AP50	AP75
RX-FPN			59.64	81.8	79.16	67.61	84.4	81.14
RX-PAFPN	✔		62.82	88.08	82.71	69.46	90.56	82.78
PR-RX-FPN		✔	67.35	88.6	84.88	75.13	90.87	87.95
MPAPR R-CNN	✔	✔	68.6	90.78	85.31	75.98	91.19	89.15

✔ indicates that on the basis of the base network, add the corresponding module at ✔. The first line is the base network (RX-FPN). The second line is the base network+PAFPN, abbreviated as RX-PAFPN. The third line is the base network+PointRend, abbreviated as PR-RX-FPN. The fourth line is the base network+PAFPN+PointRend, abbreviated as MPAPR R-CNN.

Meanwhile, as for the PointRend, the AP_seg and AP_box score of PR-RX-FPN is significantly improved by 7.71% (from 59.64% to 67.35%) and 7.52% (from 67.61% to 75.13%). The result demonstrates that the PointRend has more influence than PAFPN. This is because a uses both coarse and fine prediction of points and fuses the two features, which is more effective for canopy edges detection. Combined with PAFPN and PoitRend, MPAPR R-CNN obtained the most excellent canopy detection and segmentation results with AP_seg and AP_box score improved by 8.96% (from 59.64% to 68.6%) and 8.37% (from 67.61% to 75.98%), respectively. Therefore, MPAPR R-CNN is more effective for canopy detection task.

**Figure 9:**
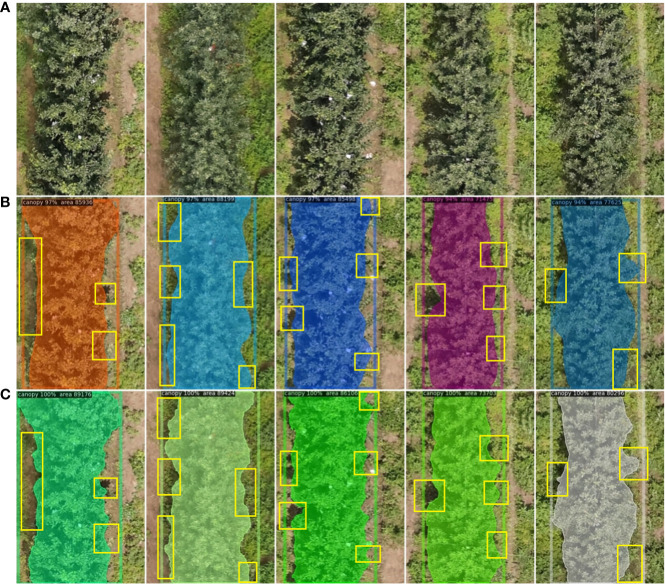
Some examples of canopy images of interferences. **(A)** Input of the detected raw image. **(B)** Mask loss curves. **(C)** Mask point loss curves.Yellow rectangular boxes indicate details with significantly different test results.

Examples of the results of several methods for canopy detection are shown in **Figure 9**. The good boundary segmentation performance of MPAPR R-CNN is shown in the figure by the yellow marker box. As can be shown, for the input image (**Figure 9A**), our approach (**Figure 9C**) outperforms Mask R-CNN paired with ResNeXt and FPN (**Figure 9B**) in terms of both canopy identification and segmentation. For instance, Mask R-CNN missed some edge information and incorrectly identifies the shadow of the tree as the canopy areas, but MPAPR R-CNN’s findings for detecting the canopy are more accurate. Unlike Mask R-CNN, which has a rather rough segmentation contour, MPAPR R-CNN’s segmentation contour is more defined.

### 3.3 Image stitching

**Figure 10:**
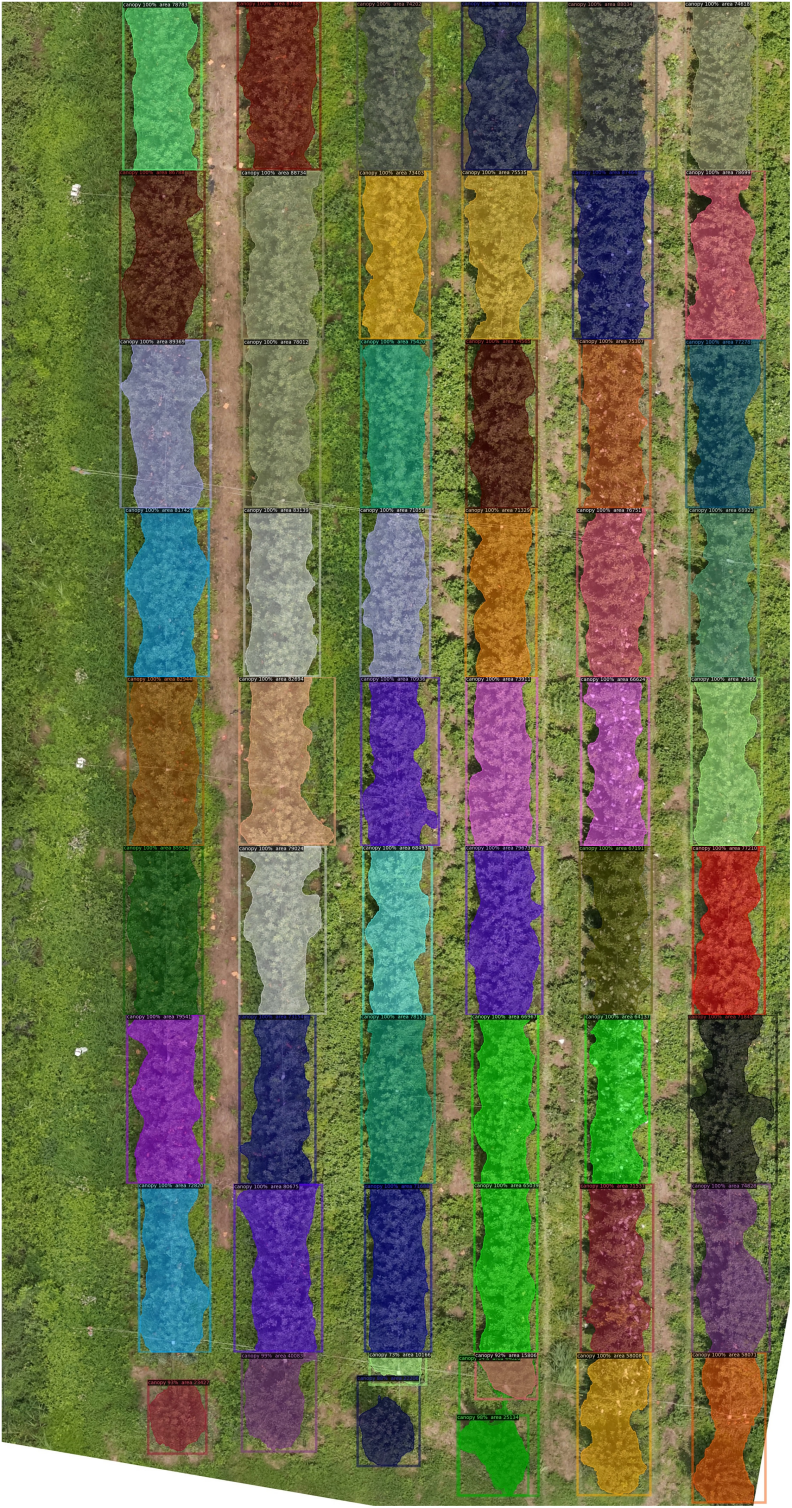
The visual results of stitching image.

After the deep learning model had been trained and the test photos had been post-identified and segmented, a high-resolution DOM map was created using Adobe Photoshop CC 2019 software. **Figures 10** shows the visual outcomes of the models. Small slices of pictures on the stitched DOM may all be inferred with accurate geo-coordinate positions, since the RGB visible camera communicates position coordinates with the UAV during image acquisition. This has ramifications for the creation of changeable application prescription maps later on.

By increasing mAP by 2.19%, our innovative segmentation method significantly improved segmentation accuracy. In the canopy detection of a mass of branches and notably for tree margins, the pixel-level target was accurately recognized. Therefore, our suggested network’s efficiency has been shown.

## 4 Discussion

### 4.1 Effect of shadows and surface vegetation on canopy edge detection is effectively solved

Most orchard canopy studies at this stage have focused on identifying the canopy of a single tree, but some researchers have also looked at methods to recognize and precisely count tree crowns with significant overlap rates. While there are many research references for techniques of geometric computation and image processing, the aforementioned two approaches are restricted to the relatively constant biological form of tree crowns and the straightforward backdrop of UAV image gathering. The geometric measuring method based on the form of the tree canopy is not reliable because the canopy shape may fluctuate significantly with the continual expansion of the tree canopy. In contrast, the instance segmentation approach might produce high performance by identifying the tree canopy’s pixels and segmenting each canopy separately with more flexibility and resilience, or inference in a unified manner. The accuracy of threshold segmentation techniques used in traditional image processing can be significantly impacted by weeds on the ground.

MPAPR R-CNN can address this issue. Firstly, we changed the original ratio of anchor frames in the RPN network. The canopy in dataset distributed along the up-down direction due to images were not rotated by 90 degrees in the data enhancement operation, the detection task of the canopy dataset better facilitated when the RPN network uses a more elongated anchor frame, such as {1:1, 1:2, 1:3}, for the generation of the region suggestion frame. In addition, performing a set of transformations using low-dimensional embeddings by constructing bases in the base block, split-transform-merge strategy can make the deep learning model learn more features, which has been effective for the problem of color interference between the surface vegetation and the canopy.

The most important thing is there are two main distinctions between the original Mask R-CNN architecture and our proposed canopy detection and segmentation approach. This is so that the RPN can generate more precise candidate boxes, which is made possible by the PAFPN module’s ability to help the backbone network gather features with rich low-level information. Furthermore, the PointRend module’s combination of coarse- and fine-grained features enhanced the segmentation accuracy of ground and canopy edges that have a comparable color palette.

**Figure 11:**
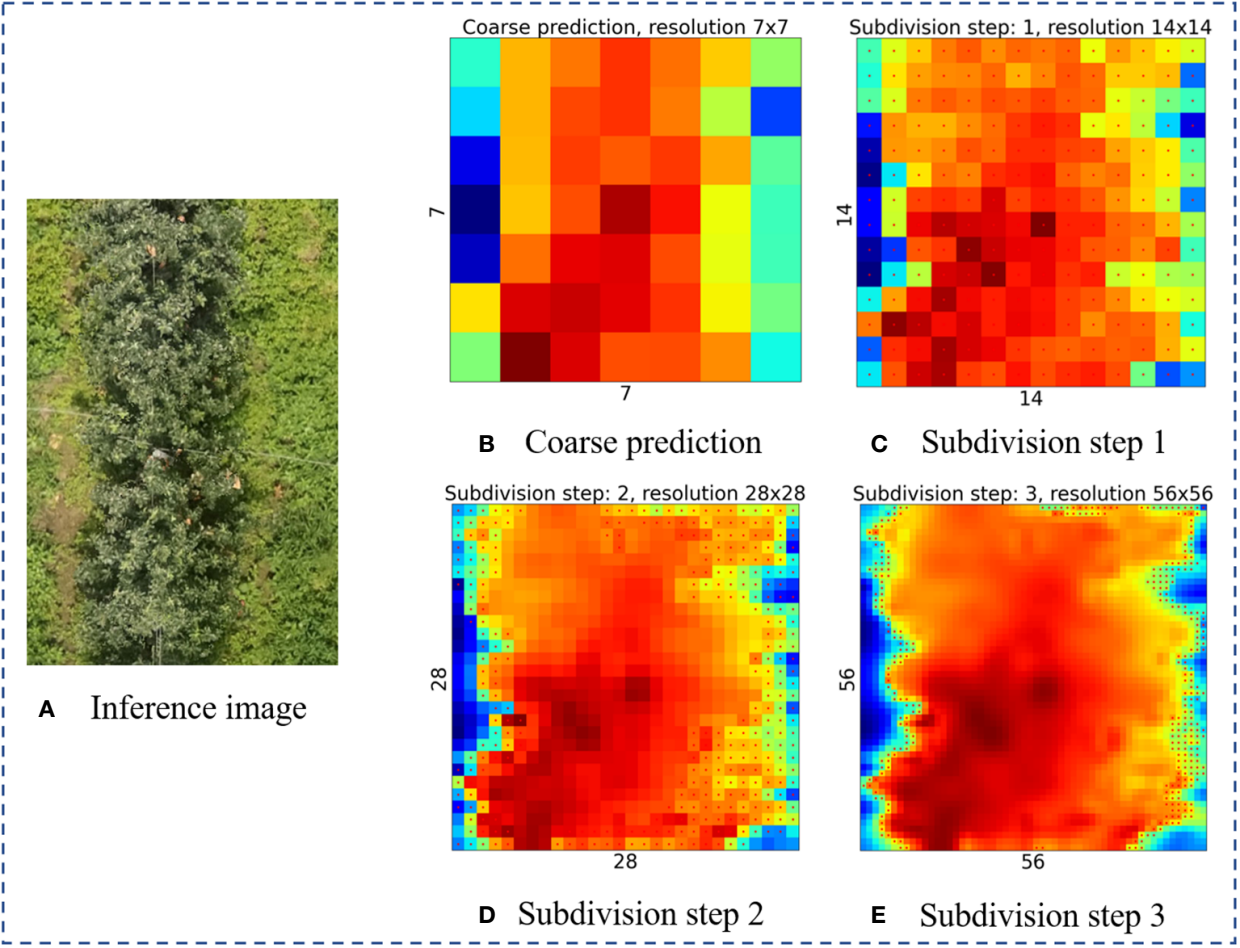
Example of inference image subdivision step. **(A)** The raw image used to visualize the inference process. **(B)** Course prediction. **(C-E)** Subdivision step 1-3, the bilinear differential upsampling is performed successively at a rate of 2x.

As shown in **Figure 11**, we visualized the process of PointRend module in canopy image inference. During the Inference process, each region is rendered by iterative coarse-to-fine. In each iteration, PointRend upsamples the previous segmentation result using bilinear differences, and then selects N uncertain points from this result. This was equivalent to purposefully selecting the N points that are difficult to segment, then extracting the feature vectors, and classifying them by MLP to get the new segmentation result, then up-sampling by a factor of 2, extracting the uncertain points, and then point prediction by MLP, and repeating this step until the prediction is completed. PointRend optimized the task of accurately recovering object edges during upsampling. Therefore, MPAPR R-CNN effectively segmented under the influence of shadows and surface vegetation and improved the recognition accuracy of canopy edges.

### 4.2 Effectiveness, limitations of UAV in orchard detectron and future work directions

The instance segmentation method enhanced by Pointrend in apple tree orchard situations was initially put out in this work. Two researchers painstakingly annotated RGB photos of the tree canopy for at least three days to create the data sets required to train deep learning models. The labeling of individual branches requires careful identification because of the severe branch crosses that result from dense planting patterns. Additionally, the canopy shadow cast by the sun on aerial photographs when it is not directly overhead presents a difficult labeling challenge. Inadequate illumination or a little swing of the drone during the photo-taking process can further degrade the picture quality of the final orthophoto image, in addition to the effects of cloudiness or wind on the UAV. More crucially, the new research on precision management in orchards has shown considerable promise for UAV gathering of photos with excellent flying efficiency. Compared with UAVs equipped with expensive multispectral or hyperspectral cameras for canopy identification methods, carrying visible light cameras is cost-effective and promising for large area applications.

We propose to focus on two topics of improvement in the upcoming work plan. First, a study may be done using the multi-spectral photos that the DJI P4 UAV captured. Multi-spectral research on canopy segmentation and individual differences in the tree canopy may be analyzed based on the chlorophyll difference between the tree canopy and ground weeds. The second is the study and development of quick and effective orchard spraying tools based on low-altitude data from UAVs on orchard distribution and canopy differences, combined with ground spraying and UAV plant protection technologies.

## 5 Conclusions

In this paper, a novel orchard canopy detection and segmentation method based on the Mask R-CNN was presented. By applying the PAFPN module and the PointRend into the original Mask R-CNN framework, combined with the improved anchor and ResNeXt, our well-trained model can automatically detect and segment canopy in orchard with high accuracy. It can be concluded that our algorithm could better capture features of the canopy edges, it could improve the accuracy of the edges of canopy segmentation results, which addressed the over- and under-sampling issues encountered in the pixel labeling tasks. It can be concluded that our algorithm could better capture features of the canopy edges, it could improve the accuracy of the edges of canopy segmentation results. Our future work will be to extend MPAPR R-CNN to many other UAV image applications.

## Data availability statement

The raw data supporting the conclusions of this article will be made available by the authors, without undue reservation.

## Author contributions

WZ collected and analyzed the data, and wrote the manuscript. XC supervised the project. JQ conceptualized the experiment, selected the algorithms, provided funding support and equipment. SY assisted in analyzing the data. All authors contributed to the article and approved the submitted version.

## Funding

This study was supported by the National Natural Science Foundation of China (31971783). Financial support from the above fund and organizations is gratefully acknowledged.

## Acknowledgments

The authors wish to thank the Jingxiang Orchard of Weihai City for the help in the collection of the ground data and field data.

## Conflict of interest

The authors declare that the research was conducted in the absence of any commercial or financial relationships that could be construed as a potential conflict of interest.

## Publisher’s note

All claims expressed in this article are solely those of the authors and do not necessarily represent those of their affiliated organizations, or those of the publisher, the editors and the reviewers. Any product that may be evaluated in this article, or claim that may be made by its manufacturer, is not guaranteed or endorsed by the publisher.
